# Using linkage to assess coverage of population estimates

**DOI:** 10.23889/ijpds.v9i5.2556

**Published:** 2024-09-18

**Authors:** Josie Plachta

**Affiliations:** 1 The Office For National Statistics, UK

## Objectives

The Demographic Index (DI) comprises of five linked administrative datasets, used for population estimation. Current linkage methods are not ideal to utilise the power of this asset. Using the 2021 England and Wales Census, we developed an innovative composite linkage method to fully utilise the power of the DI.

## Approach

Using non-greedy deterministic and probabilistic linkage methods, we linked the DI to the Census at a composite level where we believe links exist – i.e., linking a Census record with a DI cluster (consisting of linked records from the data sources used to make the DI). Next, pairwise linkage of records within these clusters was performed to ensure that every DI record within the cluster was a link. Clerical review of a sample of the full linkage was performed using clerical resolution and search to resolve uncertain and conflicting links and to inform the quality of our linkage.

Due to data quality errors in the administrative data, clerical techniques were not always able to determine true match status, so clerical identified ‘uncertain’ links to estimate precision and recall ranges rather than a set estimate.

## Results

A quality analysis to estimate linkage accuracy was conducted and suggests that the linkage has a precision of between 99.3% and 99.7%, and recall between 99.1% and 99.7%.

## Conclusions

We have linked the ONS’ composite population-level dataset to the 2021 England and Wales Census. The presentation will showcase the methods developed and how we ensured the highest quality possible.

